# Bifurcation-based dynamics and internal resonance in micro ring resonators for MEMS applications

**DOI:** 10.1007/s11071-025-11379-7

**Published:** 2025-06-08

**Authors:** Saber Azizi, Hamed Haddad Khodaparast, Hadi Madinei, Mohammad I Younis, Ghader Rezazadeh

**Affiliations:** 1 Aerospace Department, Faculty of Science and Engineering, Swansea University, Swansea, UK; 2 Department of Mechanical Engineering, State University of New York, Binghamton, NY 13902 USA; 3 Centre for Materials Technologies, Skolkovo Institute of Science and Technology, Moscow, Russia; 4 Mechanical Engineering Department, Faculty of Engineering, Urmia University, 11km Sero Road, Urmia, 16557153 Iran

**Keywords:** Nonlinear dynamics, Internal resonance, Micro ring, MEMS, Duffing oscillator, Bifurcation

## Abstract

This paper presents a novel investigation into the dynamics of a micro ring structure subjected to harmonic base excitation, designed as a highly sensitive MEMS mass sensor or bifurcation-based switch. Leveraging the in-plane nature of the motion, the system exhibits an exceptionally low damping ratio, making it ideal for detecting subtle changes in dynamic behaviour. The governing nonlinear differential equations, incorporating the geometric nonlinearities of the support beams, were derived and simplified into a reduced-order model consisting of coupled nonlinear Duffing-type equations. A key innovation of this study lies in the tunability of the system’s frequency ratios, enabling the activation of a 1:3 internal resonance. By varying the length of the support beams while keeping the central ring geometry fixed, the first two natural frequencies were carefully examined, revealing a significant influence on the dynamic response. Frequency response curves confirmed the presence of 1:3 internal resonance near the primary resonance of the first mode, highlighting the potential for efficient energy transfer between modes. Furthermore, a detailed bifurcation analysis uncovered a range of complex nonlinear phenomena, including nonlinear modal interactions, torus bifurcations, quasi-periodic motion, and cyclic fold bifurcations. These bifurcations not only provide deeper insight into the system’s dynamics but also offer additional operational mechanisms for switching applications. The findings demonstrate the system’s capability to exploit nonlinear dynamics for enhanced sensitivity and robustness, paving the way for the development of next-generation MEMS sensors and bifurcation-based devices.

## Introduction

Exploring nonlinear dynamics and internal resonance has led to numerous applications, including energy harvesting, vibration control, metamaterials, and MEMS/NEMS actuators and sensors [1–3]. Internal resonance in nonlinear dynamics, specifically the interaction between modes with specific frequency ratios, has been extensively studied by multiple researchers [4]. Key contributions were made by Nathan Newmark [5] and Rosenberg [6] in their studies of coupled oscillations. Generally, the interaction between multiple vibrational modes through internal resonance provides a pathway to achieve increased sensitivity, broaden operational frequency ranges, and improve energy efficiency, making it a promising approach for developing next-generation technologies.

Extensive research has been conducted into the fundamental study of internal resonance in various nonlinear dynamic systems. This includes but is not limited to, self-excited systems, parametric excitation, controlling the location of isolated response curves, frequency locking, multiple internal resonance couplings, and quasi-periodicity patterns [7–14]. However, several studies have also focused on the utilisation of internal resonance properties in real-world applications. One important application is vibration-based energy harvesting, where several studies have demonstrated the advantages of increased sensitivity and a broadened frequency range by exploiting internal resonance. Ardakani et al. [15] explored the dynamic coupling between a rectangular container’s pendular oscillations and internal fluid sloshing, highlighting its application in ship roll motion and wave energy converters, where fluid mode interactions impact system performance. Xing et al. [16] studied a multi-directional piezoelectric energy harvester inspired by body hair, utilising internal resonance to achieve energy harvesting from multiple directions, thereby broadening the system’s bandwidth. Similarly, Fan et al. [17] demonstrated how nonlinear modal interactions in a piezoelectric energy harvester, even without magnetic nonlinearities, could enhance energy transfer and operational bandwidth.

Internal resonance has also shown significant potential in metamaterials and composites. Failla et al. [18] introduced a locally resonant metamaterial structure with periodic resonator arrays, creating multiple band gaps by exploiting internal resonance. Placidi et al. [19] explored similar bandgap structures using tensegrity prisms, demonstrating how internal resonance can reduce bandgap frequencies through rotational motion. Moreover, the internal resonance concept has shown great potential in vibration control and attenuation. Hu et al. [20] investigated internal resonance in a spatial flexible beam suspended by springs within a tethered system, demonstrating that internal resonance enhances attitude stability and accelerates energy transfer, leading to improved vibration control. Su et al. [21] investigated a nonlinear cable-stayed beam system with a tuned mass damper (TMD), focusing on one-to-one internal resonance between the beam, cable, and TMD during the primary resonance of the beam. Xiong et al. [22] proposed a vibration isolation system based on a bio-inspired X-shaped structure with a nonlinear absorber, focusing on the system’s nonlinear dynamics under 3:1 internal resonance. They showed that adjusting structural parameters, such as rod length and assembly angle, can effectively control the system’s nonlinearity and improve vibration isolation. Further research into nonlinear absorbers was conducted by Shami et al. [23], who experimentally validated a nonlinear piezoelectric shunt absorber that achieves 2:1 internal resonance, leading to amplitude saturation and effective vibration attenuation.

Internal resonance has also shown great potential for improving MEMS, particularly in their application as actuators and sensors [14]. The review paper by Hajjaj et al. [24] provides an overview of recent advances in micro and nanoresonators. MEMS resonators have been widely employed for detecting ultra-minute particles across various applications, including antibiotic detection [25], vaccinia virus characterization [26], and xenon atom detection [27]. In terms of utilising internal resonance in MEMS actuators and sensors, Rahmanian and Awrejcewicz [28] developed an electrostatic MEMS actuator leveraging 2:1 internal resonance, showing how energy transfer between bending and torsional modes can generate frequency combs with enhanced power and spacing. Xia et al. [29] proposed a double amplification scheme for mass sensitivity in non-Duffing internal resonance systems with even power nonlinearities. Zamanzadeh et al. [30] investigated internal resonances in a levitation force MEMS actuator with flexible cantilever and clamped-clamped microbeam configurations, showing that Von Karman nonlinearity plays a key role in enabling internal resonance by bringing vibration modes closer together and enhancing energy exchange. Grenat et al. [31] explored the global dynamics of an array of electrostatically coupled nonlinear MEMS resonators, focusing on symmetry breaking and localisation phenomena using nonlinear normal modes, nonlinear forced response curves, and bifurcation analysis. They demonstrated that symmetry breaking and motion localisation in the MEMS array can be leveraged for mass detection, with the sensitivity of this method adjustable through bias voltage, mass distribution, or geometry modification. Recent studies have also highlighted the role of higher-order internal resonances, such as 1:3 and 3:1 modal interactions, in improving MEMS sensor performance. Wang and Ren [32] demonstrated how three-to-one internal resonance in MEMS arch resonators enhances nonlinear interactions, which can be utilized for highly sensitive mass detection. Similarly, Houri et al. [33] investigated the occurrence of 1:3 internal resonance in MEMS sensors, showing how mode coupling can amplify small perturbations induced by added mass. Kumar et al. [34] explored the dynamic behavior of electrostatically actuated microbeams undergoing 3:1 internal resonance, revealing its potential for frequency-based mass sensing. The nonlinear modal interactions observed in these systems suggest that carefully tuning MEMS devices to operate near specific internal resonance conditions can significantly enhance their detection sensitivity and robustness to external noise. Additionally, Ouakad et al. [35] examined both one-to-one and three-to-one internal resonances in MEMS shallow arches, demonstrating how initial rise and mid-plane stretching influence nonlinear modal coupling. Their study suggests that engineering these modal interactions can further improve MEMS resonators’ mass sensing capabilities by increasing sensitivity and selectivity to external perturbations. Furthermore, Guillot et al. [36] analyzed an experimental setup where 1:3 internal resonance was used in a beam with piezoelectric patches, demonstrating a strong correlation between the added mass and the frequency shift induced by internal resonance dynamics. These findings indicate that leveraging higher-order internal resonances can provide a novel approach to improving mass sensing accuracy in MEMS devices.

In this paper, we investigate the dynamics and bifurcation analysis of a ring, representing the structure of a novel configuration for a potential mass sensor, which utilises in-plane vibration with low damping. The ring is supported by four beams attached to fixed supports, and the entire structure is subjected to harmonic base excitation. We examine the potential occurrence of internal resonance and analyse the different bifurcation types. The occurrence of internal resonance is particularly significant, as it leads to a substantial increase in the signal-to-noise ratio near higher modes, which is crucial for sensing applications in MEMS. Besides analysing the frequency response curves, a bifurcation analysis is also conducted, in which various bifurcation types are identified, with particular emphasis on torus bifurcation. The bifurcation diagrams and the associated Poincaré sections are developed by numerically integrating the associated differential equations.

## Modelling

**Fig. 1:**
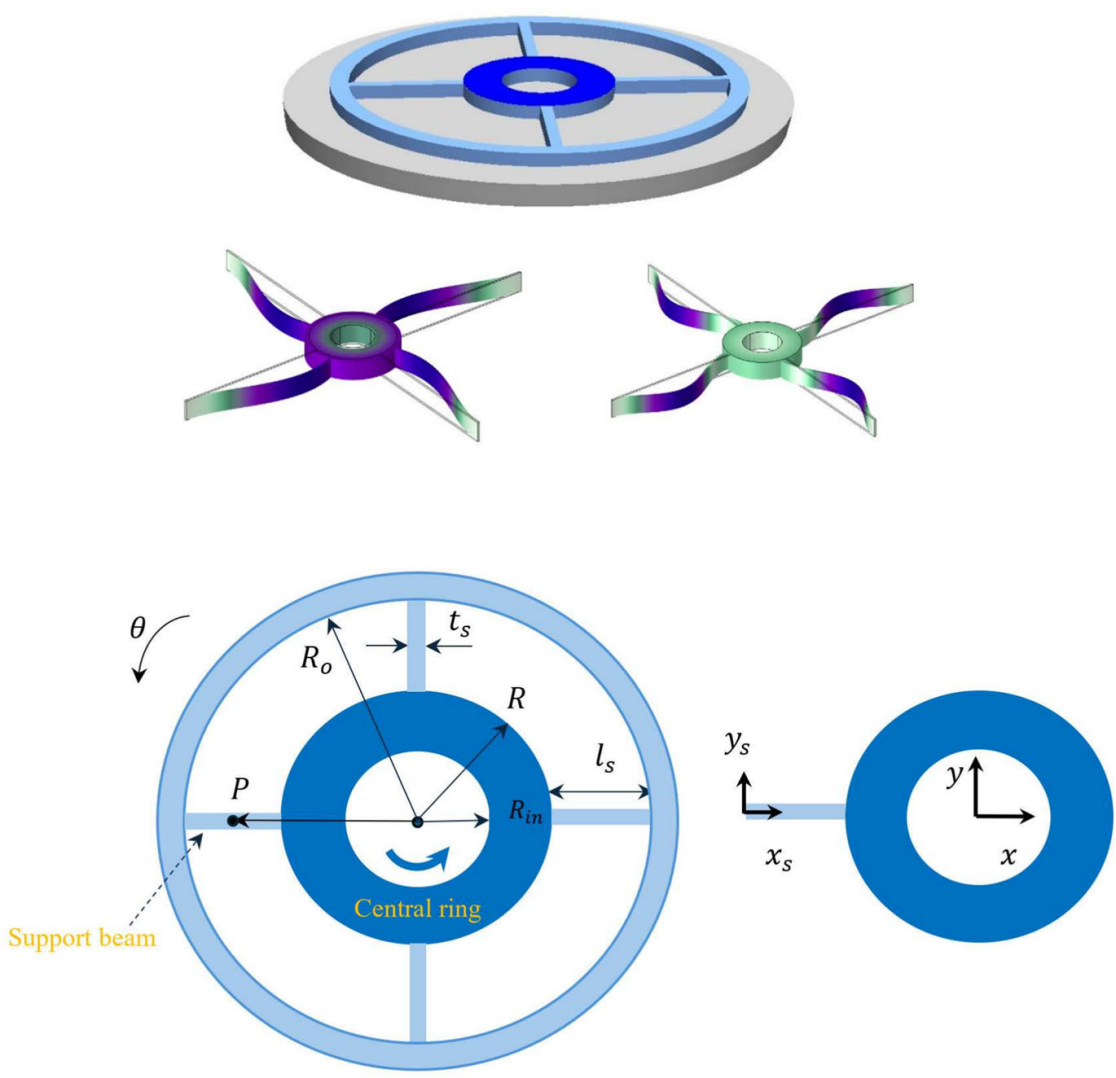
Schematics of the central ring and the support beams with the applied coordinate systems $x_s$-$y_s$ and *x*-*y* subjected to base excitation $\theta$, with the first two mode shapes included (by COMSOL)

As illustrated in Figure 1, the model consists of a central ring with an outer radius *R* and an inner radius $R_in$, connected to the substrate by four supporting beams of length $l_s$, thickness *h*, and width $t_s$. The coordinate system $x_s - y_s$ is attached to the left end of the support beam. Harmonic base excitation is applied in the form of $\theta (t) = \theta _0 \sin (\omega t)$ about the centre of the ring. The material has a density $\rho$ and Young’s modulus *E*. The coordinate system $x - y$ is attached to the centre of the ring and rotates with it.

In the *x*-*y* coordinate system, with unit vectors $\textbf{i}$ and $\textbf{j}$ attached to the centre of the ring, the position vector $\vec {R}_p$ of a point *p* on the support beam can be expressed as:

1 $$\begin{aligned} \vec {R}_p = (-R_o + x_s) \textbf{i} + w_s \textbf{j} \end{aligned}$$

where $w_s$ is the lateral deflection measured along $y_s$. Considering the harmonic base excitation $\theta (t)$, and the unit vectors of the rotary coordinate system *x*-*y* which we have used to express $\vec {R_p}$ the velocity of point *p* can be expressed as follows:

2 $$\begin{aligned} \dot{\vec {\textbf{R}}}_p = \frac{d}{dt} \left[ (-R_o + x_s) \textbf{i} + w_s \textbf{j} \right] _{\text {rot}} + \dot{\theta }(t) \vec {\textbf{k}} \times \vec {\textbf{R}}_p \end{aligned}$$

Eq. (2) is then simplified to:

3 $$\begin{aligned} \dot{\vec {R}}_p = -\dot{\theta }(t) w_s \textbf{i} + \left( \dot{w}_s + \dot{\theta }(t)(-R_o + x_s) \right) \textbf{j} \end{aligned}$$

Considering Eq. (3), and assuming the central ring to be rigid, the kinetic energy of the system can be expressed as follows:

4 $$\begin{aligned} T= &   \frac{1}{2} (\rho A)_s n_s \int _0^{l_s} \left( \left( -\dot{\theta } w_s\right) ^2 \right. \nonumber \\  &   \left. + \left( \dot{w}_s + \dot{\theta } (-R_o + x_s) \right) ^2 \right) dx_s + \frac{1}{2} I_D (\dot{\varphi } + \dot{\theta })^2\nonumber \\ \end{aligned}$$

where, $I_D$ represents the mass moment of inertia of the disc, including the added mass, about its centre. The term $\dot{\varphi }$ denotes the angular velocity of the disc relative to the base, and $n_s$ is the number of support beams. Assuming the stretching effect and neglecting longitudinal inertia, the potential energy of the support beams can be expressed as follows [37]:

5 $$\begin{aligned} U = \frac{1}{2} (EI)_s n_s \int _0^{l_s} w_s''^2 dx_s + \frac{EA}{8l_s} n_s \left( \int _0^{l_s} w_s'^2 dx_s \right) ^2 \end{aligned}$$

Here, *I* and *A* represent the second moment of inertia about the neutral axis and the cross-sectional area of the support beams, respectively. Assuming the solution takes the form $w_s(x_s, t) = \sum _{i=1}^{n} q_i(t)\psi _{si}(x_s)$, where $q_i(t)$ and $\psi _{si}(x_s)$ are the generalised coordinates [38–40] and mode shapes that satisfy the boundary conditions for the support beams. The mode shapes are derived based on the fact that the system includes support beams and a tip mass, which in this case is the central ring. Due to the symmetry of the problem and the fact that the longitudinal displacements of the support beams correspond to very high frequencies, it is assumed that the central ring undergoes no translational displacement. The details of the mode shape derivation are provided in Appendix B. The kinetic and potential energies simplify to:

6 $$\begin{aligned} T&= \frac{1}{2} (\rho A)_s n_s \left( \dot{\theta }^2 \sum _{i=1}^{n} \sum _{j=1}^{n} q_{i}(t) q_{j}(t) \right. \nonumber \\&\quad \left. \int _0^{l_s} \psi _{s_{i}}(x_s) \psi _{s_{j}}(x_s) dx_s \right. \nonumber \\&\quad + \sum _{i=1}^{n} \sum _{j=1}^{n} \dot{q}_{i}(t) \dot{q}_{j}(t) \int _0^{l_s} \psi _{s_{i}}(x_s) \psi _{s_{j}}(x_s) dx_s \nonumber \\&\quad + \dot{\theta }^2 \int _0^{l_s} (R_o - x_s)^2 dx_s \nonumber \\&\quad \left. - 2\dot{\theta } \sum _{i=1}^{n} \dot{q}_{i}(t) \int _0^{l_s} (R_o - x_s) \psi _{s_{i}}(x_s) dx_s \right) \nonumber \\&\quad + \frac{1}{2} I_D \left( \sum _{i=1}^{n} \sum _{j=1}^{n} \dot{q}_{i}(t) \dot{q}_{j}(t) \psi '_{s_{i}}(l_s) \psi '_{s_{j}}(l_s) \right. \nonumber \\&\quad \left. + \dot{\theta }^2 + 2\dot{\theta } \sum _{i=1}^{n} \dot{q}_{i}(t)\right) , \nonumber \\ U&= \frac{1}{2} (EI)_s n_s \left( \sum _{i=1}^{n} \sum _{j=1}^{n} q_i(t) q_j(t) \right. \nonumber \\&\quad \left. \int _0^{l_s} \psi ''_{s_{i}}(x_s) \psi ''_{s_{j}}(x_s) dx_s \right) \nonumber \\&\quad + \frac{EA}{8 l_s} n_s \left( \sum _{i=1}^{n} \sum _{j=1}^{n} \sum _{k=1}^{n} \sum _{o=1}^{n} q_i(t) q_j(t) q_k(t) q_o(t)\right. \nonumber \\&\quad \left. \int _0^{l_s} \psi '_{s_{i}}(x_s) \psi '_{s_{j}}(x_s) dx_s \int _0^{l_s} \psi '_{s_{k}}(x_s) \psi '_{s_{o}}(x_s) dx_s \right) \end{aligned}$$

In Eq. (6), the angular velocity of the central ring with respect to the base ($\dot{\varphi }$) is approximated by $\sum _{i=1}^{n} \psi '_{si}(l_s) \dot{q}_i(t)$, which represents the gradient of the support beams at $x_s = l_s$. (As mentioned, the central ring is assumed to be rigid, so its angular velocity is linked to the time rate of change of the slope at the tip of the support beams.)

To account for the effect of dissipation on the equations of motion, Rayleigh’s dissipation function is defined as follows [41]:

7 $$\begin{aligned} R_D = \frac{1}{2} c_s n_s \int _0^{l_s} \left( \frac{\partial w_s}{\partial t} \right) ^2 dx_s \end{aligned}$$

Here, $c_s$ is the damping coefficient per unit length of the support beam. Considering the non-dimensionalising parameters $T_t$, *g*, and $\Theta$, the non-dimensional parameters $\hat{t}$, $\hat{w}_s$, and $\hat{\theta }$ are defined as follows:

8 $$\begin{aligned} \hat{t} = \frac{t}{T_t}, \quad \hat{w}_s = \frac{w_s}{g}, \quad \hat{\theta } = \frac{\theta }{\Theta }, \quad \hat{x}_s = \frac{x_s}{l_s} \end{aligned}$$

where,

$$\begin{aligned} {T_t}=\sqrt{\frac{\rho A l_s^4}{{(EI)}_{s}}} \end{aligned}$$

To simplify the notation, the overhead hats are omitted for the remainder of the paper. By incorporating Eq. (8) into the Lagrangian function $(L = T - U)$, and considering the contribution of the first two modes in the solution, the equations of motion simplify to:

9 $$\begin{aligned}&M_1 \ddot{q}_1 + M_2 \ddot{q}_2 + K_{l_1} q_1 + K_{l_2} q_2 + K_{n_1} q_1^3 \nonumber \\&\quad + K_{n_2} q_2^3 + K_{g_1} q_1 q_2^2 + K_{g_2} q_2 q_1^2 \nonumber \\&\quad + C_1 \dot{q}_1 + C_2 \dot{q}_2 = F_1 \nonumber \\&M_2 \ddot{q}_1 + M_3 \ddot{q}_2 + K_{l_2} q_1 + K_{l_3} q_2 \nonumber \\&\quad + K_{n_3} q_1^3 + K_{n_4} q_2^3 + K_{g_3} q_1 q_2^2 + K_{g_4} q_2 q_1^2 \nonumber \\&\quad + C_2 \dot{q}_1 + C_3 \dot{q}_2 = F_2 \end{aligned}$$

**Table 1:** Mechanical and geometrical properties of the ring and the support beams

**Parameter**	**Value**
$l_s$	300 $\mu$m
$R_o$	190 $\mu$m
*R*	40 $\mu$m
$R_{in}$	20 $\mu$m
*h*	20 $\mu$m
*E*	112.4 GPa
$t_s$	2 $\mu$m
$n_s$	4
$\rho$	2330 kg/m$^3$

**Fig. 2:**
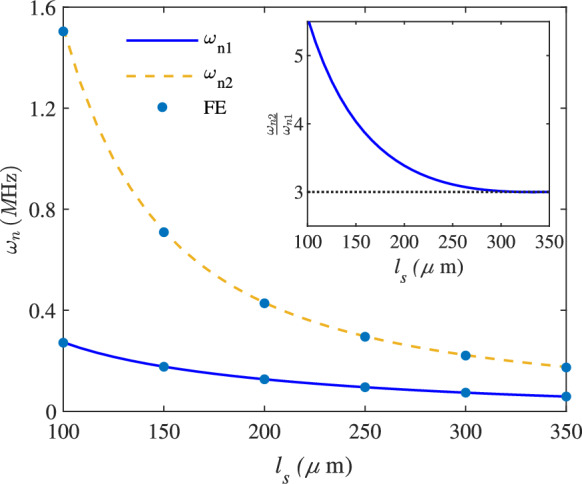
Variation of the first two natural frequencies of the model with the length of the support beams, with the inset showing the frequency ratios, FE stands for the Finite Element analysis

**Fig. 3:**
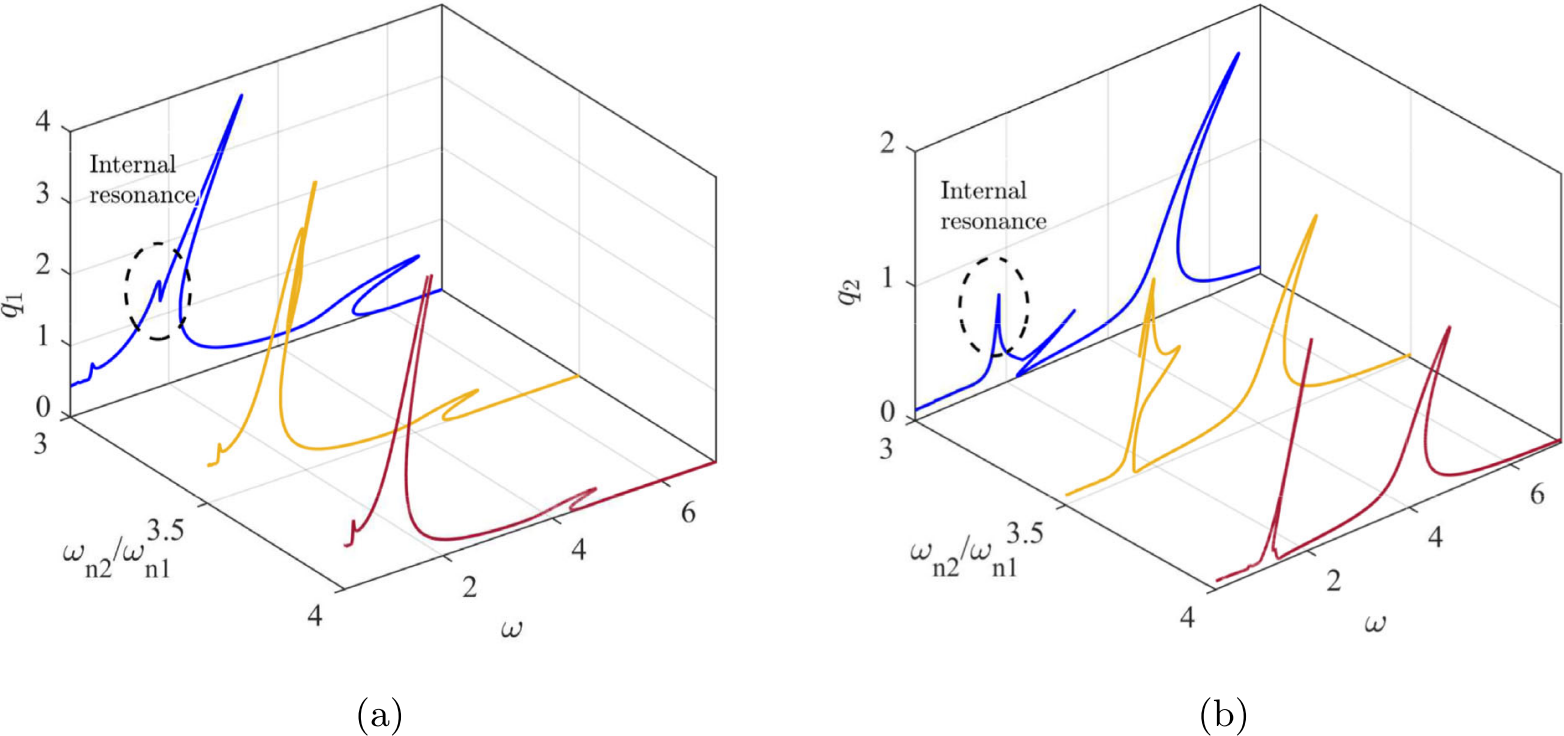
Frequency response curves associated with (a): First mode, (b) Second mode for three different values of $\omega _{n2} / \omega _{n1}$

**Fig. 4:**
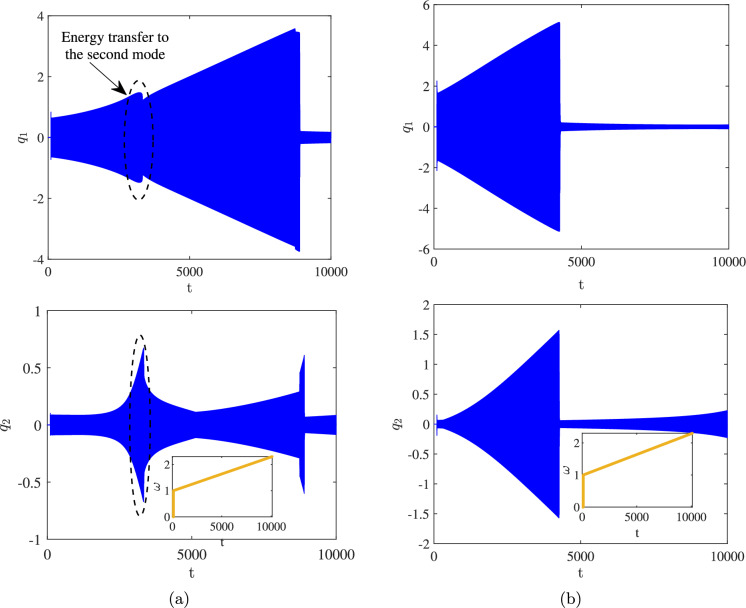
Frequency sweep and the associated time response for the first and second mode with different frequency ratios: (a) $\frac{\omega _{n2}}{\omega _{n1}} = 3$, (b) $\frac{\omega _{n2}}{\omega _{n1}} = 4$, where the frequency sweep is shown as an inset

In Equation 9 The parameters $M_1$ , $M_2$ , $M_3$ are the mass coefficients, $K_{l_1}$ , $K_{l_2}$ , $K_{l_3}$ are the coefficients of the linear stiffness, $K_{n_1}$ , $K_{n_2}$ , $K_{n_3}$ are the coefficients of the non-linear qubic stiffness, $K_{g_1}$ , $K_{g_2}$ , $K_{g_3}$ are the coefficients of the non-linear quadratic stiffness, and $C_1$ , $C_2$ , $C_3$ are the coefficients of the damping terms. These coefficients are introduced in Appendix B.

To numerically integrate the equations of the motion (Eq. 9), the associated phase space variables have been introduced and numerically integrated using the ODE45 in Matlab which is based on the Runge Kutta method.

The frequency response curves are obtained through numerical continuation of periodic solutions in forced nonlinear dynamical systems. The system is typically described by a set of first-order differential equations [42]:

10 $$\begin{aligned} \dot{\textbf{x}} = \textbf{f}(\textbf{x}, \omega , F) \end{aligned}$$

where $\textbf{x}$ represents the state variables, $\omega$ is the excitation frequency, and $F$ is the forcing amplitude. Periodic solutions $\textbf{x}(t) = \textbf{x}(t + T)$ are computed using collocation or shooting methods. The continuation process systematically varies $\omega$ while solving for steady-state periodic responses. Stability is determined using Floquet theory, where the eigenvalues $\mu _i$ of the monodromy matrix $\textbf{M}$ indicate stability if

11 $$\begin{aligned} |\mu _i| < 1. \end{aligned}$$

By tracing stable and unstable branches, the frequency response curves are constructed, and the key nonlinear phenomena such as resonance, bifurcations, and jump discontinuities are captured [37].

## Results and Discussions

Table 1 summarises the key parameters used in the model, including geometric dimensions, material properties, and other relevant constants for the micro-ring under study.

**Fig. 5:**
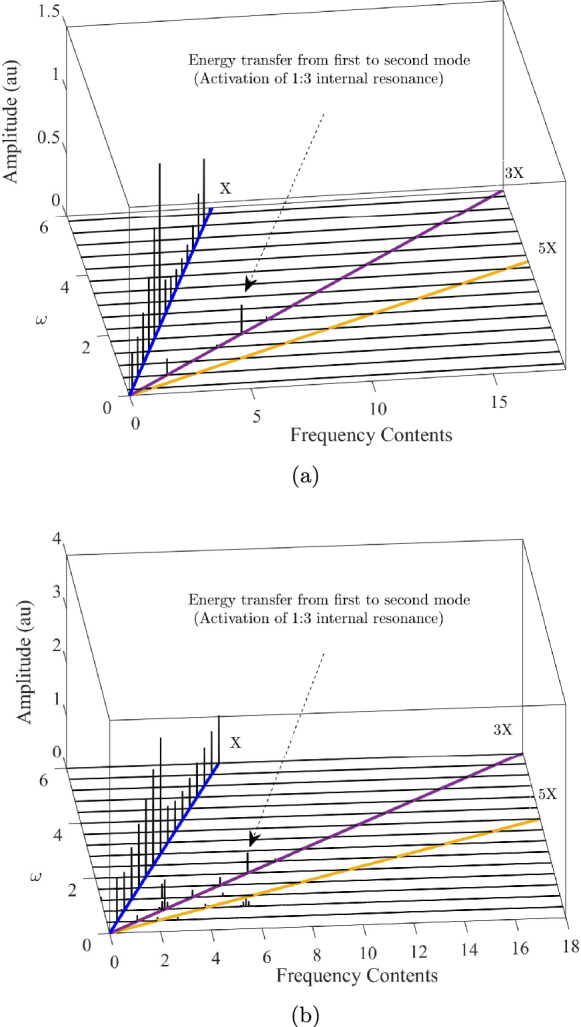
Spectrum cascade for two different base excitation amplitudes: (a) $\theta _0 = 1$, (b) $\theta _0 = 4$ (*X*,3*X* and 5*X* multiples of the excitation frequency *X* have been separately displayed), ’au’ represents an arbitrary unit

In order for internal resonance to be activated, we need to determine the specific geometry at which the ratio of the natural frequencies matches the order of the dominant nonlinearity in the system. Therefore, we have investigated the effect of $l_s$ on the first two natural frequencies and their ratio. Figure 2 illustrates the effect of the support beam length, $l_s$, on the first two natural frequencies, $\omega _{n1}$ and $\omega _{n2}$, of the device. The blue dots are representing the results of the finite element analysis (FE).

As illustrated, increasing the length of the support beams results in a reduction of both the first and second natural frequencies. However, the rate of reduction for the second frequency is significantly greater than that of the first, leading to a decreased gap between the two frequencies. This effect has been utilised as a frequency ratio tuning mechanism in this study. The results have been compared with finite element analysis conducted in COMSOL, demonstrating excellent agreement. The frequency ratio of $\frac{\omega _{n2}}{\omega _{n1}}$ with respect to the variation of $l_s$ is displayed as an inset in 2. For $l_s = 300$ $\mu \text {m}$, the frequency ratio reaches a value of 3, which has been selected as the support beam length in this study. This ratio enables the occurrence of internal resonance within the system. Figure 3 displays the frequency response curves for the first two modes at three different values of $\omega _{n2}/\omega _{n1}$. The excitation frequency $\omega$ is varied from below the primary resonance to values just above the second mode.

As demonstrated, for a frequency ratio of 4, the amplitudes of both modes exhibit a significant increase near the resonance frequencies, characterised by a hardening response. This behaviour is attributed to the geometric nonlinearity of the support beams, which induces a hardening effect. Reducing the frequency ratio from 4 to 3 facilitates energy transfer between the two modes. When the system is excited near the primary resonance, energy is transferred from the first mode to the second, resulting in a significant increase in the amplitude of the second mode. This phenomenon is analysed and justified based on the frequency content of the time response presented in the subsequent results. Figure 4 shows the time response of the system for two different frequency ratios, 3 and 4, as $\omega$ is swept at a very low rate from 1 to 2, encompassing the primary resonance in between. The variation of the excitation frequency with respect to time is shown in the inset. Considering the time constants associated with the resonance frequencies ($\omega _{n1} = 1.66$ and $\omega _{n2} = 5.00$), the frequency sweep rate of 1/1000 is sufficiently low to ensure that the solution settles into the attractor corresponding to each individual excitation frequency.

As shown, for the frequency ratio $\omega _{n2} / \omega _{n1} = 3$, while the frequency is swept in the vicinity of the primary resonance, modal interaction occurs, allowing energy to be transferred from the first mode to the second. Consequently, a decrease in the amplitude of the first mode and an increase in the amplitude of the second mode are observed. In the remainder of the paper, the presented results are based on $\omega _{n2} / \omega _{n1} = 3$, which corresponds to $l_s = 300 , \mu m$.

**Fig. 6:**
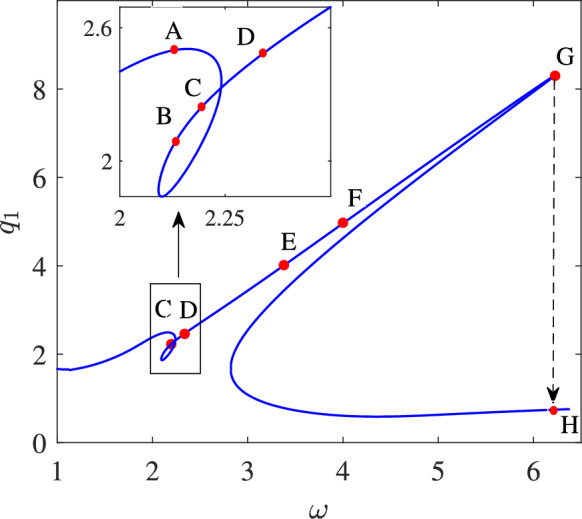
Frequency response curves associated with $\theta _0 = 4$

Figure 5 displays the spectrum cascade as the excitation frequency is varied from 0.5 to 6. This range encompasses the first two resonance frequencies and extends just above the second mode. As given in Appendix A, the base excitation is assumed to take the form $\theta = A\sin (\omega t)$, where the amplitude *A* is defined such that the angular acceleration remains constant as the excitation frequency varies. This is achieved by setting $A = \omega _0^2 \theta _0 / \omega ^2$, where $\omega _0$ and $\theta _0$ are constants representing the starting frequency and the amplitude of rotation at $\omega = \omega _0$.

**Fig. 7:**
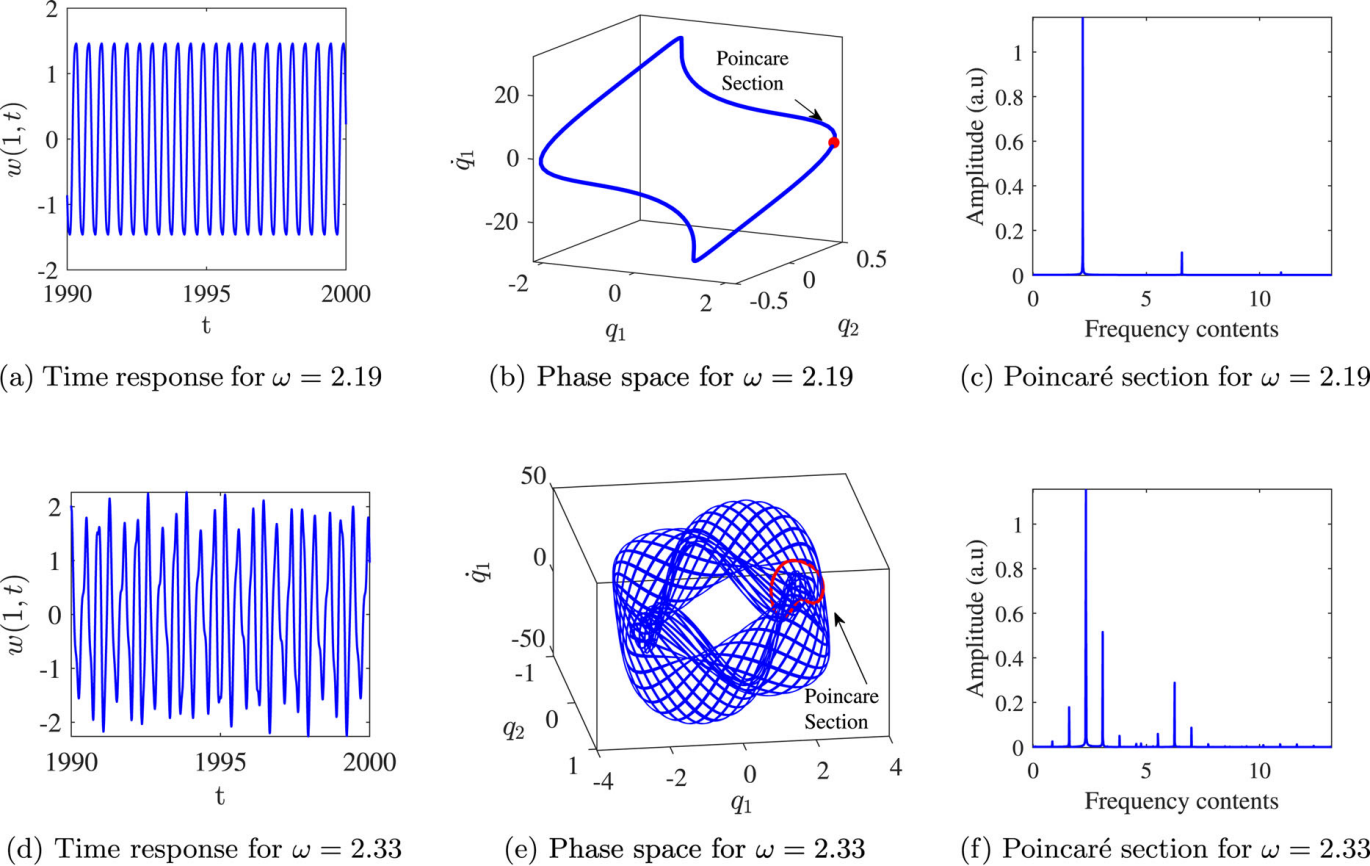
Time response, phase space, and Poincaré section for $\theta _0 = 4$, $\omega _0 = 0.2$

As illustrated in Figure 5, the horizontal axes represent the excitation frequency ($\omega$) and the corresponding frequency components of the response, while the vertical axis shows amplitude. We varied $\omega$ from 0.5 to 6, slightly beyond the second resonance frequency. For the case where $\theta _0 = 1$, the response frequency components align primarily along the *X* axis, indicating that the response occurs mainly at the excitation frequency. However, at two specific frequencies, highlighted in Figure 5a, additional behaviour emerges. Near $\omega = 0.5$, a superharmonic resonance indirectly excites the primary resonance. This occurs due to the tripling of the excitation frequency, which brings it within the primary resonance zone. Once this point is passed, the 3*X* component diminishes, leaving the *X* component to dominate the response. This pattern continues until $\omega = 1.8$, where the 3*X* component reappears, triggered by the frequency-tripling mechanism that aligns the 3*X* component with the resonance zone of the second mode. This interaction facilitates energy transfer to the second mode, resulting in another peak along the 3*X* line. This phenomenon is significant for bifurcation-based switches that operate on internal resonance principles, as it demonstrates a notably lower signal-to-noise ratio for the second mode compared to the first. This behaviour can be advantageous for applications such as MEMS mass sensors, where it serves as an effective sensing mechanism. Figure 5b shows the spectrum cascade for the case $\theta _0 = 4$. This scenario corresponds to a high-amplitude response, resulting in increased complexity due to the activation of additional nonlinear frequency components beyond the 3*X* component. Notably, even in this case, modal interaction occurs in the vicinity of the primary resonance, and the contribution of the second mode to the response significantly increases.

**Fig. 8:**
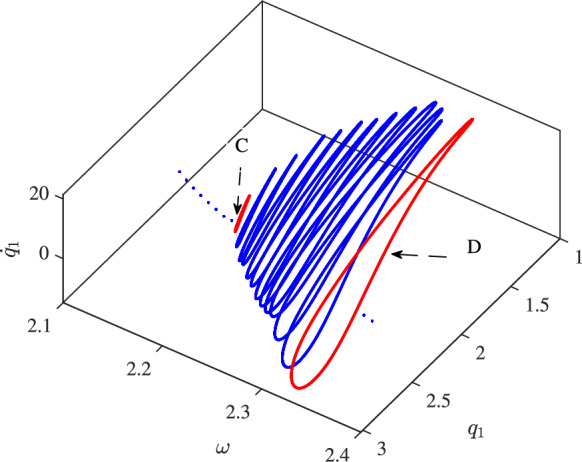
Development of the Poincaré sections of the response as the frequency is swept from before point C to past point D. The Poincaré sections associated with the secondary Hopf bifurcations are highlighted in red

**Fig. 9:**
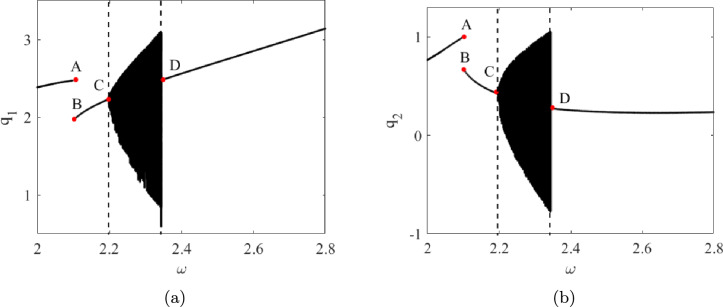
Bifurcation diagram associated with $\theta _{0}=4$, $\omega _{0}=0.2$

Figure 6 shows the frequency response curve associated with the amplitude of the first mode for the case $\theta _0 = 4$ and $\omega _0 = 0.2$.

The bifurcation points $(C, D, E, F, \text {and } G)$ are marked on the frequency response curve. At points *C* $(\omega = 2.19)$ and *D* $(\omega = 2.33)$, the system undergoes two torus bifurcations, where a pair of complex conjugate Floquet multipliers move away from the unit circle [43, 44]. This phenomenon, also known as a Neimark-Sacker bifurcation or secondary Hopf bifurcation, indicates the onset of quasi-periodic behaviour. At points *E* and *F*, the system experiences two Branch Point Cycle (BPC) bifurcations at point *E* $(\omega = 3.38)$ and *F* $(\omega = 4.00)$, while a cyclic fold bifurcation [42] occurs at point *G*, where the Floquet multipliers exit the unit circle along the real axis in the complex plane. This latter bifurcation is catastrophic in nature, as the system exhibits a sudden drop when the frequency exceeds point *G*, transitioning to point *H*. Such bifurcations can be exploited as a switching mechanism, particularly in bifurcation-based switches.

We focus on secondary Hopf bifurcations, as they can potentially serve as operational regions for bifurcation-based MEMS switches. To examine the type of response before and after the secondary Hopf bifurcation, we have investigated the time response in the vicinity of points *C* and *D*. The corresponding time response, phase space, and Poincaré section are illustrated in Figure 7.

As shown in Figure 7, just before bifurcation point C, the system exhibits a periodic response with frequency components in commensurate ratios, leading to a single equilibrium point on the Poincaré section as the time response is sampled at the excitation period. Between points C and D, the system transitions to quasi-periodic motion with incommensurate frequency ratios, resulting in a limit cycle on the Poincaré section. This transition of the equilibrium point to a limit cycle on the Poincaré section resembles the bifurcation of an equilibrium point into a limit cycle on the phase space (Hopf bifurcation), which occurs in the digitised response and is thus termed a Secondary Hopf Bifurcation. The appearance of this bifurcation in the system potentially enables an additional switching mechanism, particularly useful in bifurcation-based switches. The development of the Poincaré sections of the response from before bifurcation point C through to beyond point D is depicted in Figure 8. This figure shows the system’s behaviour as it transitions from periodic motion, represented by a single point on the Poincaré section before point C, to quasi-periodic motion, characterised by a limit cycle beyond point D.

The corresponding bifurcation diagram associated with $q_1$ and $q_2$ is shown in Figure 9. Points A, B, C, and D correspond to those depicted in Figure 6.

Starting the frequency sweep from the left of point A, the system exhibits periodic motion at the excitation frequency or its higher harmonics. This is evident as the sampling at the excitation frequency intersects the Poincaré section only once. As the frequency is swept forward, the response jumps to the lower branch periodic orbit at point B, continuing to exhibit similar periodic motion. Further sweeping of the excitation frequency results in a pitchfork bifurcation at point C, where the response becomes quasi-periodic, leading to an infinite number of intersections with the Poincaré section. This response is distinguished from chaotic motion, as the associated frequency content qualitatively rules out chaotic behaviour.

## Conclusion

This study explored the nonlinear dynamics and bifurcation behaviour of a ring microstructure, designed as a novel in-plane vibrating configuration for a potential MEMS mass sensor with low damping. The equations of motion were derived and discretised into a reduced-order model, represented by coupled Duffing-type equations with cubic nonlinearity. The influence of the supporting beam geometry on the ratios of the first two natural frequencies was thoroughly examined, revealing that both frequencies decrease with increasing beam length, albeit at different rates, with a more pronounced reduction observed for the second resonance frequency. A key outcome of this investigation was the identification of a specific geometry where the ratio of the first two resonance frequencies equalled 3, enabling the activation of a 1:3 internal resonance. This feature is particularly novel, as it facilitates efficient energy transfer between modes, enhancing the sensor’s signal-to-noise ratio and sensitivity. Frequency response curves confirmed the occurrence of internal resonance, and the associated energy transfer between the first two modes was clearly established. This dynamic behaviour is crucial for improving the performance of the proposed mass sensor, especially in scenarios where the second mode significantly contributes to the system’s response. Additionally, spectral cascades were presented for varying excitation amplitudes, illustrating the energy transfer from the first to the second mode near the primary resonance. At higher base excitation amplitudes, nonlinear superharmonic resonance zones emerged, further expanding the sensor’s dynamic range and operational capabilities. Bifurcation analysis revealed several critical bifurcations, including Secondary Hopf (torus bifurcations), branch points of cycles, and cyclic fold bifurcations, identified through the loci of Floquet multipliers associated with the limit cycles. These bifurcations are of particular importance as they introduce additional switching mechanisms, offering operational flexibility for the sensor. Special attention was given to the Hopf bifurcation points, with time responses, Poincaré sections, and bifurcation diagrams providing deeper insights into the system’s behaviour. Between torus bifurcation points, the system exhibited quasi-periodic motion due to incommensurate frequency components, with the transition from an equilibrium point to a limit cycle clearly demonstrated in the Poincaré sections. The findings of this study highlight the potential of this novel in-plane vibrating MEMS mass sensor configuration, offering enhanced performance through tailored nonlinear dynamic behaviour and bifurcation-driven mechanisms. These insights pave the way for the design and optimisation of next-generation bifurcation-based MEMS sensors and switches.

## Figures and Tables

**Fig. 10:**
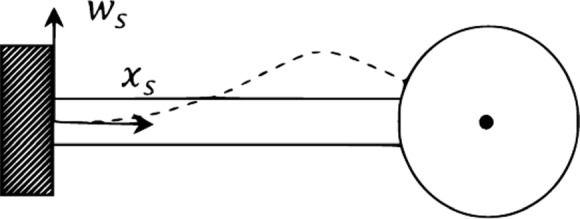
Schematics of a disc with a pin at the centre connected to a clamped beam

## Data Availability

No datasets were generated or analysed during the current study.

## Appendix A

In this part we have derived the shape functions associated with the model. Assuming a disc, pinned at the centre, with radius $$R$$, connected to a beam with length $$l_s $$,

**Table 2:** Coefficients for equation terms

$M_1 = 2\alpha _{1_{11}} + 2\alpha _{2_{11}}$	$M_2 = \alpha _{1_{21}} + \alpha _{2_{21}} + \alpha _{1_{12}} + \alpha _{2_{12}}$
$K_{l_1} = 2\beta _{1_{11}} - 2\Gamma _{1_{11}}$	$K_{l_2} = \beta _{1_{21}} + \beta _{1_{12}} - \Gamma _{1_{21}} - \Gamma _{1_{12}}$
$K_{n_1} = 4\beta _{2_{1_{111}}}$	$K_{n_2} = \beta _{2_{2_{221}}} + \beta _{2_{2_{212}}} + \beta _{2_{2_{122}}} + \beta _{2_{1_{222}}}$
$C_1 = 2\gamma _{11}$	$K_{g_1} = 2\beta _{2_{1_{221}}} + 2\beta _{2_{1_{112}}} + 2\beta _{2_{1_{212}}} + 2\beta _{2_{2_{111}}} + 2\beta _{2_{1_{211}}}$
$C_2 = \gamma _{12} + \gamma _{21}$	$K_{g_2} = 3\beta _{2_{1_{112}}} + 3\beta _{2_{1_{111}}} + 3\beta _{2_{1_{211}}} + 3\beta _{2_{1_{121}}}$
$$F_1 = -\dot{\Gamma }_{2_{1}} - \dot{\Gamma }_{3_{1}}$$	
$M_3 = 2\alpha _{1_{22}} + 2\alpha _{2_{22}}$	$K_{l_3} = 2\beta _{1_{22}} - 2\Gamma _{1_{12}}$
$K_{n_3} = \beta _{2_{1_{122}}} + \beta _{2_{1122}}$	$K_{g_3} = 2\beta _{2_{1121}} + 3\beta _{2_{2211}}$

The kinetic (*T*) and potential (*U*) energies will be in the following forms:

A1 $$\begin{aligned} \begin{aligned} T&= \frac{\rho A}{2} l_s \left( \frac{g}{T_t} \right) ^2 \int _{0}^{1} \dot{w}_s^2 \, dx_s\\&\quad + \frac{1}{2} I_D \left( \frac{g}{T_t l_s} \right) ^2 \left( \frac{\partial \dot{w}_s}{\partial x} (1,t) \right) ^2, \\ U&= \frac{(EI)_s}{2} \left( \frac{g}{l_s^2} \right) ^2 \int _{0}^{1} \left( \frac{\partial ^2 w_s}{\partial x_s^2} \right) ^2 l_s \, dx_s \end{aligned} \end{aligned}$$

Non-dimensional parameters $\hat{x} = x_s/l_s$, $\hat{w}_s = w_s/g$, $\hat{t} = t/T_t$ have been substituted for $x_s$, $w_s$, and *t* respectively. For simplicity, the hats have been dropped. Here $l_s$, *g* and $T_t$ are non-dimensionalising parameters. In Eq. A1, the rotation of the disc is approximated by the slope of the beam at the connection point to the disc, i.e., ${w_s}_x(1,t) = \varphi$. Applying integration by parts, the variation of the Hamiltonian is set to zero:

**Table 3:** Coefficients of the Terms in the System Equations

$\alpha _{1_{ij}} = \frac{1}{2} n_s (\rho A)_s \frac{g^2 l_s}{T^2} \int _0^1 \psi _{s_i}(x_s) \psi _{s_j}(x_s) dx_s$	$\gamma _{ij} = \frac{1}{2} n_s c_s \frac{g^2 l_s}{T^2} \int _0^1 \psi _{s_i}(x_s) \psi _{s_j}(x_s) dx_s$
$\alpha _{2_{ij}} = \frac{1}{2} I_D \frac{g^2}{l_s^2 T^2} \psi '_{s_i}(1) \psi '_{s_j}(1)$	$\Gamma _{1_{ij}} = \frac{1}{2} n_s (\rho A)_s \dot{\theta }^2 \frac{g^2 \Theta ^2 l_s}{T^2} \int _0^1 \psi _{s_i}(x_s) \psi _{s_j}(x_s) dx_s$
$\beta _{1_{ij}} = \frac{1}{2} n_s (EI)_s \frac{g^2}{l_s^3} \int _0^1 \psi ''_{s_i}(x_s) \psi ''_{s_j}(x_s) dx_s$	$\Gamma _{2_i} = -n_s (\rho A)_s \dot{\theta } \frac{\Theta g l_s}{T^2} \int _0^1 (R_o - l_s x_s) \psi _{s_i}(x_s) dx_s$
$\theta = A \sin (\omega t) \quad A = \omega _0^2 \theta _0 / \omega ^2$	$\Gamma _{3_i} = I_D \dot{\theta } \frac{\Theta g}{T^2 l_s} \psi '_{s_i}(1)$
$$\beta _{2ijko} = \frac{EA g^4}{8 l_s^3} n_s \int _0^1 \psi '_{s_i}(x_s) \psi '_{s_j}(x_s) dx_s \int _0^1 \psi '_{s_k}(x_s) \psi '_{s_o}(x_s) dx_s$$	

A2 $$\begin{aligned}&\int _0^t \left\{ \int _0^1 \left( -\rho A l_s \left( \frac{g}{T_t} \right) ^2 \ddot{w}_s \delta w_s - (EI)_s \frac{g^2}{l_s^3} w_s^{IV} \delta w_s \right) dx_s \right. \nonumber \\&\quad + \left( -I_D \left( \frac{g}{T_tl_s} \right) ^2 {\ddot{w}_{s,x}}(1,t) \delta {w_{s,x}}(1,t) - EI \frac{g^2}{l_s^3} {w_s}_{xx} \delta {w_s}_x \Big |_{0}^{1} \right. \nonumber \\&\quad \left. \left. + EI \frac{g^2}{l_s^3} {w_s}_{xxx} \delta w_s \Big |_{0}^{1} \right) \right\} dt = 0 \end{aligned}$$

Assuming $T_t^2 = \frac{\rho A l^4}{EI}$, the linear motion equation and the associated boundary conditions reduce to:

A3 $$\begin{aligned}  &   \ddot{w} + w^{IV} = 0-I_D \left( \frac{g}{{T_t}{l_s}} \right) ^2 \ddot{w}_{sx}(1,t) \delta {w_s}_x(1,t)\nonumber \\  &   \quad - (EI)_s \frac{g^2}{l_s^3} {w_s}_{xx}(1,t) \delta {w_s}_x(1,t)\nonumber \\  &   \quad + (EI)_s \frac{g^2}{l_s^3} {w_s}_{xxx}(1,t) \delta {w_s}(1,t)\nonumber \\  &   \quad + (EI)_s \frac{g^2}{l_s^3} {w_s}_{xx}(0,t) \delta w_x(0,t) \nonumber \\  &   \quad - (EI)_s \frac{g^2}{l^3} w_{xxx}(0,t) \delta w(0,t) = 0 \end{aligned}$$

With the assumption $\delta w_s(1,t) = -\frac{R}{l_s} \delta {w_s}_x(1,t)$, the boundary condition terms in Eq. A3 reduce to:

A4 $$\begin{aligned}  &   I_D \left( \frac{g}{T_tl_s} \right) ^2 \ddot{w}_{sx}(1,t) \frac{l_s}{R}\nonumber \\  &   \quad + (EI)_s \frac{g^2}{l^3} {w_s}_{xxx}(1,t) + (EI)_s \frac{g^2}{l^3} {w_s}_{xx}(1,t) \frac{l}{R} = 0 \end{aligned}$$

A5 $$\begin{aligned}  &   {w_s}(0,t) = 0, \quad {w_s}_x(0,t) = 0, \quad {w_s}(1,t) = -\frac{R}{l_s} \nonumber \\ \end{aligned}$$

The first two shape functions which satisfy the boundary conditions specified in Eq. A4 have been derived as follows:

A6 $$\begin{aligned} \psi _1(x_s)= &   2.8743 \sin (1.8958x_s) \nonumber \\  &   - 2.0588 \cos (1.8958x_s) \nonumber \\  &   - 2.8743 \sinh (1.8958x_s) \nonumber \\  &   + 2.0588 \cosh (1.8958x_s) \end{aligned}$$

A7 $$\begin{aligned} \psi _2(x_s)= &   1.0015 \sin (4.8111x_s) \nonumber \\  &   - 1.0177 \cos (4.8111x_s) \nonumber \\  &   - 1.0015 \sinh (4.8111x_s) \nonumber \\  &   + 1.0177 \cosh (1.8958x_s) \end{aligned}$$

## Appendix B

The coefficients of the terms in Eq. 9 are as follows:
